# Prevalence and Risk Factors of Workplace Violence Against Emergency Department Nurses in China

**DOI:** 10.3389/ijph.2022.1604912

**Published:** 2022-08-04

**Authors:** Zihui Lei, Shijiao Yan, Heng Jiang, Jing Feng, Shuyang Han, Chulani Herath, Xin Shen, Rui Min, Chuanzhu Lv, Yong Gan

**Affiliations:** ^1^ Department of Social Medicine and Health Management, School of Public Health, Tongji Medical College, Huazhong University of Science and Technology, Wuhan, China; ^2^ Department of Emergency Medicine, Hunan Provincial People’s Hospital/The First Affiliated Hospital, Hunan Normal University, Changsha, China; ^3^ School of Public Health, Hainan Medical University, Haikou, China; ^4^ Centre for Alcohol Policy Research, School of Psychology and Public Health, La Trobe University, Melbourne, VIC, Australia; ^5^ Melbourne School of Population and Global Health, University of Melbourne, Melbourne, VIC, Australia; ^6^ The Fourth Clinical College, Zhejiang Chinese Medical University, Hangzhou, China; ^7^ Department of Psychology and Counselling, Faculty of Health Sciences, The Open University of Sri Lanka, Colombo, Sri Lanka; ^8^ Emergency Medicine Center, Sichuan Provincial People’s Hospital, University of Electronic Science and Technology of China, Chengdu, China; ^9^ Research Unit of Island Emergency Medicine, Chinese Academy of Medical Sciences, Hainan Medical University, Haikou, China

**Keywords:** emergency department nurses, workplace violence, prevalence, influencing factors, China

## Abstract

**Objectives:** We aim to find out the prevalence, characteristics, and predictors of workplace violence (WPV) against current Chinese emergency department (ED) nurses.

**Methods:** A cross-sectional survey of 20,136 ED nurses was conducted in 31 provinces in China between July and September 2019. Descriptive analyses were used to examine the prevalence and characteristics of WPV. Chi-square analysis and Binary logistic regression analysis were used to identify the predictors of WPV.

**Results:** During the past 12 months, there are 79.39% of ED nurses exposed to any type of WPV, including 78.38% and 39.65% exposed to nonphysical and physical violence, respectively. Binary logistic regression analysis shows that ED nurses who were male, had bachelor’s degrees, had average monthly salary between 5,001 and 12,000, worked in central China, had higher professional titles, were more experienced, arranging shift work, and had higher work stress were more likely to experience WPV.

**Conclusion:** A relatively high prevalence of WPV against Chinese ED nurses has been revealed in this study. The characteristics and predictors of WPV remind us to take positive measures to reduce WPV.

## Introduction

Workplace violence (WPV) is defined as incidents where the staff was abused, threatened, or assaulted in circumstances related to their work with explicit or implicit challenges to staff safety, well-being, or even health [1]. Violence and aggression against staff have been documented as a significant problem for healthcare workers (HCWs) [2]. According to a survey from America in 2015, HCWs were approximately four times higher to require time off due to violence than all other private-sector employees [3]. Moreover, rates of non-fatal workplace injuries due to WPV against HCWs were 5–12 times higher than rates for US workers overall [4]. Emergency department (ED) nurses, worked in an environment where multiple environmental risk factors exist, such as long waiting times, understaffed department and unrestricted movement of the public, and had more frequent contact with patients or patients’ families, which caused a higher rate of WPV among other HCWs [5,6]. Moreover, a meta-analysis covering 81,771 Chinese healthcare professionals concluded that ED was the most vulnerable department exposed to WPV in hospitals, and nurses had higher rates than physicians [6].

According to previous researches, the 12-month prevalence of WPV against ED workers was 31.0% (95% CI, 26.0%–36.0%) for physical violence and 62.3% (95% CI, 53.7%–70.8%) for nonphysical violence [7]. And the 12-month prevalence of ED nurses was 49.5% (95% CI, 19.7%–89.0%) for physical violence and 81.3% (95% CI, 40.0%–100%) for nonphysical violence [8]. All above revealed a grim situation of WPV against ED nurses. Besides, WPV inflicts multiple impacts on nurses both physically and psychologically, and leads to reduced job satisfaction, poor work performance, high nurse turnover and even poor quality of life, which will initiate a destructive impact on not only personal health but also organizational efficiency [9,10]. In addition, the prevalence of WPV in hospitals was often underestimated attributable to low reporting rates [6,11,12], the actual rates may be much higher. Considering the severe state of WPV, clarifying the influencing factors of WPV against ED nurses will facilitate a good working environment for them.

Large-scale studies about HCWs have already been established [13,14], and prospective studies [15,16] and longitudinal studies [17,18] as well. However, only small-scale cross-sectional studies, sample size was less than 500, about ED nurses were done in China and abroad [5,19]. Therefore, a large-scale and thorough research on WPV and its predictors among Chinese ED nurses is a necessity. The purpose of this research is to explore the prevalence, characteristics, and predictors of WPV against ED nurses in a large Chinese national sample.

## Methods

### Participants and Sampling

A cross-sectional study was conducted from July 2019 to September 2019 in 31 provinces across China. Samples in this study were selected by multistage stratified sampling. Firstly, we classified 31 provinces into high-developed, medium-developed, and less-developed by per capita disposable income. Secondly, 10 hospitals were randomly selected from each province. Finally, a 30% proportion of nurses with at least 6 months of experience in EDs were selected. In total, 21,912 ED nurses were requested to participate in this survey. Online questionnaires were distributed to all participants through WeChat, and local investigators in 31 provinces took responsibility for the questionnaire collection. In review, 544 nurses did not respond, 631 questionnaires were discarded because of demographic information missing, and 601 were discarded for work tenure less than 6 months in EDs. Eventually, 20,136 questionnaires were eligible for analysis.

The study protocol was approved by the Institutional Ethics Board of the Second Affiliated Hospital of Hainan Medical University, Haikou, China. All individuals provided written informed consent.

### Instrument and Measurement

The questionnaire was designed based on literature reviews, group discussions, and mock interviews. And a pilot study was conducted in a community hospital in Wuhan to evaluate the quality of the questionnaire. The self-designed questionnaire included eight sections: 1. Socio-demographic information; 2. Work-related variables; 3. Life quality and behavior habits; 4. Attitude towards pre-hospital first aid; 5. WPV scale and WPV-related questions; 6. Center for Epidemiological Studies-Depression (CES-D); 7. Maslach Burnout Inventory General Survey (MBI-GS); 8. Turnover Intention. According to the research purpose, four sections were explored: 1. Socio-demographic information includes age, gender, marital status, education level, geographic region, average monthly salary, and socioeconomic development level. 2. Work-related variables: contract status, professional title, hospital level, ownership, work tenure, shift work, and work stress. 3. WPV scale and WPV-related questions, all questions related to the prevalence, reasons, reactions and characteristics of WPV. 4. Life quality and behavior habits: self-perceived health status, history of hypertension, history of diabetes, history of coronary heart disease (CHD), alcohol drinking, smoking, exercise, and sleep quality. For more convenient dissemination, the paper questionnaire was transformed into an online questionnaire through the Questionnaire Star platform.

### Workplace Violence Scale

Workplace Violence Scale developed by Wang et al. [20] was based on Chinese national conditions, and it showed good reliability and validity to measure the prevalence of WPV against medical staff in China [21]. This scale includes five questions, and each question has four options ranging from 0′ (none) to 3′ (more than 3 times per year). Physical assault, verbal abuse, threat, verbal sexual harassment, and sexual assault were measured respectively. In this study, the Cronbach’s alpha of the scale is 0.793, and the KMO value is 0.768, which means relatively good reliability and validity. In addition, to further explore the differences between physical and nonphysical violence, these five questions were classified as physical violence (i.e., physical assault and physical sexual assault) and nonphysical violence (i.e., verbal abuse, threat, and verbal sexual harassment) by their characteristics.

### Data Collection and Quality Control

Questionnaire Star platform that produced our online questionnaire can timely feedback on the participants’ completion state. And our repeated feedback to local investigators during the surveying period assured the high responsibility. For preventing repeat filling, each device was only allowed to submit once. Finally, the original data was entered into a professional statistic software database by a professor to ensure accuracy.

### Data Analysis

The descriptive analysis used frequency and percentage for qualitative data, means and standard deviation for quantitative data, all quantitative data with severe skewness were transformed into qualitative data for analysis. The chi-square test was used to identify variables that were significantly associated with WPV. Any type of WPV, nonphysical violence, and physical violence were taken as the dependent variables, respectively, and the sociodemographic factors, work-related factors, and life quality and habits were taken as independent variables, to establish a multivariable logistic stepwise regression model and to explore influencing factors of WPV against ED nurses. The significance level for all statistical analyses was *p* < 0.05 using a two-tailed test and SPSS 22.0 was used to analyze the survey data.

## Results

The majority of these 20,136 ED nurses were women (89.41%), were married (66.19%), had bachelor’s degrees (65.99%), had an average monthly salary between 5,001 and 12,000 (54.28%), had a temporary contract (76.72%), had an elementary and lower professional title (73.36%), worked in governmental hospitals (95.90%) and tertiary hospitals (74.30%). The mean age of participants was 30.83 (SD = 6.42, ranging from 18 to 65 years). Geographically, more participants were from eastern China (38.03%) and western China (36.05%). Besides, high-developed regions (33.29%) and medium-developed regions (39.45%) had more participants than low-developed regions (27.26%). Other detailed information has shown in Table 1.

**TABLE 1:** Distributions of characteristics of workplace violence in emergency department nurses (China, 2019).

Variables	Total n (%)	Any type of WPV* n (%)	Physical violence n (%)	Non-physical violence n (%)
Total	20,136 (100.00)	15,985 (100.00)	7,984 (100.00)	15,782 (100.00)
Demographic variables				
Age (years)				
18–29	9,824 (48.79)	7,594 (47.51)	3,736 (46.79)	7,477 (47.38)
30–44	9,309 (46.23)	7,638 (47.78)	3,899 (48.84)	7,561 (47.91)
≥45	1,003 (4.98)	753 (4.71)	349 (4.37)	744 (4.71)
Gender				
Male	2,133 (10.59)	1766 (11.05)	1,176 (14.73)	1747 (11.07)
Female	18,003 (89.41)	14,219 (88.95)	6,808 (85.27)	14,035 (88.93)
Marital status				
Unmarried	6,318 (31.38)	4,908 (30.70)	2,446 (30.64)	4,835 (30.64)
Married	13,328 (66.19)	10,676 (66.79)	5,329 (66.75)	10,551 (66.85)
Divorced	461 (2.29)	379 (2.37)	202 (2.53)	374 (2.37)
Widowed	29 (0.14)	22 (0.14)	7 (0.09)	22 (0.14)
Education level				
Associate’s degree or vocational diploma	6,735 (33.45)	4,978 (31.14)	2,407 (30.15)	4,898 (31.04)
Bachelor degree	13,288 (65.99)	10,920 (68.31)	5,539 (69.38)	10,797 (68.41)
Master degree or above	113 (0.56)	87 (0.54)	38 (0.48)	87 (0.55)
Average monthly salary (¥)				
≤5,000	8,446 (41.94)	6,534 (40.86)	3,408 (42.69)	6,432 (40.76)
5,001–12,000	10,929 (54.28)	8,853 (55.38)	4,313 (54.02)	8,757 (55.49)
>12,000	761 (3.78)	598 (3.74)	263 (3.29)	593 (3.76)
Geographic region				
Eastern China	7,657 (38.03)	6,115 (38.25)	2,892 (36.22)	6,044 (38.30)
Central China	5,224 (25.94)	4,274 (26.74)	2,262 (28.33)	4,231 (26.81)
Western China	7,255 (36.03)	5,596 (35.01)	2,830 (35.45)	5,507 (34.89)
Socioeconomic development level				
High	6,704 (33.29)	5,428 (33.96)	2,533 (31.73)	5,374 (34.05)
Medium	7,943 (39.45)	6,387 (39.96)	3,244 (40.63)	6,305 (39.95)
Low	5,489 (27.26)	4,170 (26.09)	2,207 (27.64)	4,103 (26.00)
Work-related variables				
Contract status				
Permanent	4,688 (23.28)	3,767 (23.57)	1829 (22.91)	3,730 (23.63)
Temporary	15,448 (76.72)	12,218 (76.43)	6,155 (77.09)	12,052 (76.37)
Professional title				
Elementary or below	14,771 (73.36)	11,569 (72.37)	5,818 (72.87)	11,410 (72.30)
Intermediate	4,740 (23.54)	3,914 (24.49)	1939 (24.29)	3,873 (24.54)
Senior	625 (3.10)	502 (3.14)	227 (2.84)	499 (3.16)
Hospital level				
Tertiary	14,962 (74.30)	11,987 (74.99)	6,001 (75.16)	11,847 (75.07)
Secondary or blow	5,174 (25.70)	3,998 (25.01)	1983 (24.84)	3,935 (24.93)
Ownership				
Governmental	19,310 (95.90)	15,364 (96.12)	7,656 (95.89)	15,173 (96.14)
Non-governmental	826 (4.10)	621 (3.88)	328 (4.11)	609 (3.86)
Work tenure (years)				
<10	15,138 (75.18)	11,855 (74.16)	5,908 (74.00)	11,690 (74.07)
≥10	4,998 (24.82)	4,130 (25.84)	2076 (26.00)	4,092 (25.93)
Shift work				
Yes	17,727 (88.04)	14,272 (89.28)	7,288 (91.28)	14,098 (89.33)
No	2,409 (11.96)	1713 (10.72)	696 (8.72)	1,684 (10.67)
Work stress				
Low	1,143 (5.68)	722 (4.52)	297 (3.72)	708 (4.49)
Medium	5,051 (25.08)	3,524 (22.05)	1,411 (17.67)	3,471 (21.99)
High	13,942 (69.24)	11,739 (73.44)	6,276 (78.61)	11,603 (73.52)
Life quality and behavior habits				
Self-perceived health status				
Good	7,165 (35.58)	5,104 (31.93)	2,268 (28.41)	5,026 (31.85)
General	10,201 (50.66)	8,391 (52.49)	4,206 (52.68)	8,290 (52.53)
Bad	2,770 (13.76)	2,490 (15.58)	1,510 (18.91)	2,466 (15.63)
History of hypertension				
Yes	513 (2.55)	425 (2.66)	256 (3.21)	423 (2.68)
No	19,623 (97.45)	15,560 (97.34)	7,728 (96.79)	15,359 (97.32)
History of diabetes				
Yes	236 (1.17)	196 (1.23)	129 (1.62)	193 (1.22)
No	19,900 (98.83)	15,789 (98.77)	7,855 (98.38)	15,589 (98.78)
History of CHD				
Yes	250 (1.24)	231 (1.45)	163 (2.04)	230 (1.46)
No	19,886 (98.76)	15,754 (98.55)	7,821 (97.96)	15,552 (98.54)
Alcohol drinking				
Yes	1,006 (5.00)	868 (5.43)	570 (7.14)	858 (5.44)
Quitted	364 (1.81)	296 (1.85)	168 (2.10)	291 (1.84)
No	18,766 (93.20)	14,821 (92.72)	7,246 (90.76)	14,633 (92.72)
Smoking				
Yes	765 (3.80)	649 (4.06)	449 (5.62)	643 (4.07)
Quitted	135 (0.67)	116 (0.73)	74 (0.93)	114 (0.72)
No	19,236 (95.53)	15,220 (95.21)	7,461 (93.45)	15,025 (95.2)
Exercise				
Yes	3,832 (19.03)	2,842 (17.78)	1,501 (18.80)	2,798 (17.73)
No	16,304 (80.97)	13,143 (82.22)	6,483 (81.20)	12,984 (82.27)
Sleep quality				
Good	2,536 (12.59)	1749 (10.94)	735 (9.21)	1718 (10.89)
General	10,394 (51.62)	8,034 (50.26)	3,756 (47.04)	7,927 (50.23)
Bad	7,206 (35.79)	6,202 (38.80)	3,493 (43.75)	6,137 (38.89)

∗Includes those who experienced only physical, only nonphysical, or both types of workplace violence.

WPV, workplace violence; CHD, coronary heart disease.

### Incidence of Workplace Violence

Table 2 shows the incidence of five types of violence in Chinese ED nurses in the past year. Verbal abuse (75.22%) was the most common type, followed by threat (51.51%), physical assault (37.40%), verbal sexual harassment (24.81%), and physical sexual assault (12.19%). According to the classification, 79.39% of participants had exposure to any type of WPV, 78.38% exposure to nonphysical violence, and 39.65% exposure to physical violence, respectively.

**TABLE 2:** Frequency of five types of violence against emergency department nurses (China, 2019).

Type of violence	Once n (%)	Two- or three-times n (%)	More than three times n (%)	Total n (%)
Physical assault	3,688 (18.32)	2,025 (10.06)	1,818 (9.03)	7,531 (37.40)
Verbal abuse	3,383 (16.80)	3,952 (19.63)	7,812 (38.80)	15,147 (75.22)
Threat	4,021 (19.97)	2,766 (13.74)	3,585 (17.80)	10,372 (51.51)
Verbal sexual harassment	2037 (10.12)	1,142 (5.67)	1816 (9.02)	4,995 (24.81)
Physical sexual assault	1,274 (6.33)	546 (2.71)	635 (3.15)	2,455 (12.19)

### Reasons and Characteristics of Workplace Violence

Among 15,985 ED nurses who have experienced WPV, only 12,003 participants choose to answer this part. And a general descriptive analysis was completed based on the valid responses of these participants.

According to the results stated in Table 3, the main perpetrators were the patient’s relatives, which initiated 72.40% and 74.79% of physical violence and nonphysical violence respectively. Male perpetrators (85.10% and 82.60%, for physical and nonphysical violence, respectively) far more than females. Besides, more than half of workplace violence happened during night shifts (67.31% and 62.55%, respectively), and the most frequent location of WPV was the nurse’s station (32.95% and 35.85%, respectively). Common reasons for WPV are listed as follows: unmet patient needs (56.75% and 56.53%, respectively), long waiting times (50.54% and 51.98%, respectively), drug/alcohol abuse (52.87% and 47.64%, respectively), self-perceived high medical costs (45.37% and 43.58%, respectively). Only 31.34% and 28.51% of ED nurses who experienced physical and nonphysical violence completed violence reports, 22.41% and 24.62% of physical and nonphysical violence victims took no action.

**TABLE 3:** Characteristics, reasons, and reactions to workplace violence among emergency department nurses (China, 2019).

Variables	Any type of WPV n (%)	Physical violence n (%)	Non-physical violence n (%)
Total	11,743 (100.00)	6,735 (100.00)	11,613 (100.00)
Perpetrators			
Patients	2,470 (21.03)	1,555 (23.09)	2,400 (20.67)
Patients’ relatives	8,736 (74.39)	4,876 (72.40)	8,685 (74.79)
Colleagues	73 (0.62)	48 (0.71)	72 (0.62)
Managers/Supervisors	17 (0.14)	10 (0.15)	16 (0.14)
External colleagues	26 (0.22)	18 (0.27)	26 (0.22)
General public	100 (0.85)	55 (0.82)	98 (0.84)
Visitors	151 (1.29)	91 (1.35)	150 (1.29)
Others	170 (1.45)	82 (1.22)	166 (1.43)
Gender of perpetrators			
Male	9,698 (82.59)	5,738 (85.20)	9,596 (82.63)
Female	2,045 (17.41)	997 (14.80)	2,017 (17.37)
Time of violence			
Morning shifts	2,122 (18.07)	997 (14.80)	2098 (18.07)
Afternoon shifts	2,143 (18.25)	1,123 (16.67)	2,123 (18.28)
Night shifts	7,347 (62.56)	4,533 (67.31)	7,264 (62.55)
After hours	131 (1.12)	82 (1.22)	128 (1.10)
Settings of violence			
Wards	2,714 (23.11)	1,527 (22.67)	2,659 (22.90)
Doctors’ offices	607 (5.17)	385 (5.72)	602 (5.18)
Nurse stations	4,187 (35.66)	2,219 (32.95)	4,163 (35.85)
Emergency room	3,319 (28.26)	2092 (31.06)	3,288 (28.31)
On the road from work	32 (0.27)	825 (12.25)	30 (0.26)
Others	884 (7.53)	487 (7.23)	871 (7.50)
Reasons of violence			
Long waiting time	6,069 (51.68)	3,404 (50.54)	6,037 (51.98)
Unmet patients’ need	6,609 (56.28)	3,822 (56.75)	6,565 (56.53)
Dissatisfied of doctors’ service	4,352 (37.06)	2,673 (39.69)	4,331 (37.29)
Dissatisfied of nurses’ service	3,211 (27.34)	1911 (28.37)	3,191 (27.48)
Dissatisfied of treatment effect	4,374 (37.25)	2,650 (39.35)	4,354 (37.49)
Patients’ death	1,447 (12.32)	1,004 (14.91)	1,433 (12.34)
Perpetrators’ mental disorder	1922 (16.37)	1,356 (20.13)	1870 (16.10)
Self-perceived high medical costs	5,086 (43.31)	3,056 (45.37)	5,061 (43.58)
Appealing compensation	1,699 (14.47)	1,167 (17.33)	1,689 (14.54)
Alcohol/Drug abuse	5,587 (47.58)	3,561 (52.87)	5,532 (47.64)
Others	870 (7.41)	500 (7.42)	860 (7.41)
Reactions to violence			
Took no action	2,893 (24.64)	1,509 (22.41)	2,859 (24.62)
Told friends/families	1,514 (12.89)	913 (13.56)	1,503 (12.94)
Told colleagues	4,488 (38.22)	2,514 (37.33)	4,449 (38.31)
Sought help from managers	4,567 (38.89)	2,800 (41.57)	4,525 (38.96)
Sought help from union	1,383 (11.78)	926 (13.75)	1,367 (11.77)
Sought help from police	4,204 (35.80)	2,896 (43.00)	4,153 (35.76)
Changed job	115 (0.98)	89 (1.32)	115 (0.99)
Completed the violence report	3,340 (28.44)	2,111 (31.34)	3,311 (28.51)
Prosecuted	195 (1.66)	138 (2.05)	194 (1.67)
Others	715 (6.09)	361 (5.36)	708 (6.10)

WPV, workplace violence.

### Influencing Factors of Workplace Violence

Chi-square analysis has been performed and variables that were not statistically associated with any type of WPV (contract status, history of diabetes), physical violence (contract status, ownership), and nonphysical violence (history of diabetes) were excluded from multivariable regression analysis. Binary stepwise logistic regression analysis (*ɑ*_*in*_ = 0.05, *ɑ*_*out*_ = 0.10) had been performed and results were presented in Table 4.

**TABLE 4:** Logistic stepwise regression analysis of associated factors for workplace violence against Chinese emergency department nurses (China, 2019).

Variables	Any type of WPV*^,^ ^a^	Physical violence^b^	Nonphysical violence^c^
Age (ref. 18–29 years)			
30–44	—	1.12 (1.04–1.20)	—
≥45	—	1.25 (1.06–1.48)	—
Gender (ref. Female)			
Male	1.35 (1.18–1.54)	2.03 (1.83–2.25)	1.36 (1.19–1.55)
Education level (ref. Associate’s degree or vocational diploma)			
Bachelor degree	1.39 (1.29–1.50)	1.24 (1.16–1.32)	1.38 (1.28–1.49)
Master degree or above	—	—	—
Average monthly salary (ref. >12, 000¥)			
≤5, 000	—	1.43 (1.21–1.69)	—
5, 001–12, 000	1.26 (1.04–1.52)	1.28 (1.09–1.50)	1.24 (1.03–1.50)
Geographic region (ref. Western China)			
Eastern China	0.71 (0.63–0.81)	0.84 (0.76–0.93)	0.71 (0.63–0.81)
Central China	1.20 (1.09–1.32)	1.16 (1.08–1.26)	1.22 (1.11–1.34)
Socioeconomic development level (ref. High)			
Medium	0.67 (0.59–0.76)	0.89 (0.81–0.99)	0.66 (0.58–0.74)
Low	0.49 (0.43–0.57)	0.83 (0.74–0.94)	0.48 (0.42–0.56)
Professional title (ref. Elementary or below)			
Intermediate	1.27 (1.15–1.42)	—	1.27 (1.15–1.41)
Senior	1.61 (1.27–2.03)	—	1.65 (1.31–2.08)
Work tenure (ref. <10 years)	1.22 (1.10–1.35)	1.10 (1.01–1.20)	1.23 (1.12–1.36)
Shift work (ref. No)	1.68 (1.50–1.89)	1.53 (1.38–1.70)	1.70 (1.52–1.90)
Work stress (ref. Low)			
Medium	1.15 (1.00–1.32)	—	1.15 (1.00–1.32)
High	2.13 (1.86–2.44)	1.79 (1.55–2.07)	2.10 (1.83–2.40)
Self-perceived health status (ref. Good)			
General	1.45 (1.34–1.57)	1.29 (1.20–1.38)	1.44 (1.33–1.55)
Bad	2.09 (1.80–2.41)	1.73 (1.56–1.91)	2.02 (1.76–2.33)
History of diabetes (ref. No)	—	0.69 (0.52–0.91)	—
History of CHD (ref. No)	2.12 (1.30–3.43)	1.89 (1.43–2.49)	2.15 (1.34–3.44)
Alcohol drinking (ref. No)			
Yes	1.64 (1.34–2.00)	1.51 (1.31–1.74)	1.60 (1.31–1.94)
Quitted	—	—	—
Exercise (ref. No)	0.82 (0.75–0.89)	1.10 (1.02–1.19)	0.82 (0.73–0.89)
Sleep quality (ref. Bad)			
Good	0.61 (0.54–0.69)	0.65 (0.58–0.73)	0.61 (0.54–0.69)
General	0.73 (0.67–0.79)	0.76 (0.71–0.82)	0.73 (0.67–0.80)

a 14 variables were included in the final model during stepwise regression: gender (male/female), education level (associate’s degree or vocational diploma/bachelor degree/master degree or above), geographic region (western China/central China/eastern China), socioeconomic development level (high/medium/low), average monthly salary (5,000/5,001–12,000/>12,000¥), professional title (elementary or blow/intermediate/senior), work tenure (<10/10 years), shift work (yes/no), work stress (low/medium/high), self-perceived health status (good/general/bad), history of CHD (yes/no), alcohol drinking (yes/quitted/no), exercise (yes/no), sleep quality (good/general/bad).

b 15 variables were included in the final model during stepwise regression: age (18–29/30–44/45 years), gender (male/female), education level (associate’s degree or vocational diploma/bachelor degree/master degree or above), geographic region (western China/central China/eastern China), socioeconomic development level (developed/developing/less developed), average monthly salary (5,000/5,001–12,000/>12,000¥), work tenure (<10/≥10 years), shift work (yes/no), work stress (low/medium/high), self-perceived health status (good/general/bad), history of diabetes (yes/no), history of CHD (yes/no), alcohol drinking (yes/quitted/no), exercise (yes/no), sleep quality (good/general/bad).

c 14 variables were included in the final model: gender (male/female), education level (associate’s degree or vocational diploma/bachelor degree/master degree or above), geographic region (western China/central China/eastern China), socioeconomic development level (high/medium/low), average monthly salary (≤5,000/5,001–12,000/>12,000¥), professional title (elementary or blow/intermediate/senior), work tenure (<10/≥10 years), shift work (yes/no), work stress (low/medium/high), self-perceived health status (good/general/bad), history of CHD (yes/no), alcohol drinking (yes/quitted/no), exercise (yes/no), sleep quality (good/general/bad).

* Includes those who experienced only physical, only nonphysical, or both types of workplace violence.

OR, odds ratio; CI, confidence interval; WPV, workplace violence; CHD, coronary heart disease.

According to Table 4, ED nurses who were male (odds ratio [*OR*] = 1.35), had bachelor’s degrees (*OR* = 1.39), with an average monthly salary between 5,001 and 12,000 (*OR* = 1.26), worked in central China (*OR* = 1.20), had intermediate professional title (*OR* = 1.27) and senior (*OR* = 1.61), worked tenure≥10 years (*OR* = 1.22), need to shift work (*OR* = 1.68), with medium (*OR* = 1.15) and high (*OR* = 2.13) work stress, had general (*OR* = 1.45) and bad (*OR* = 2.09) self-perceived health status, had a history of CHD (*OR* = 2.12) and drunk alcohol (*OR* = 1.64) had higher prevalence to any type of WPV. And ED nurses who worked in eastern China (*OR* = 0.71), worked in medium (*OR* = 0.67) or low (*OR* = 0.49) socioeconomic development level regions, exercised (*OR* = 0.82) and had good (*OR* = 0.61) or general (*OR* = 0.73) sleep quality were less likely exposed to any type of WPV.

In physical violence, risk factors were identified as age between 30 and 44 years (*OR* = 1.12) or ≥45 years (*OR* = 1.25), having bachelor’s degree (*OR* = 1.24), average monthly salary≤5,000 (*OR* = 1.43) or between 5,001 and 12,000 (*OR* = 1.28), working in central China (*OR* = 1.16), work tenure≥10 years (*OR* = 1.10), arranging shift work (*OR* = 1.53), having high work stress (*OR* = 1.79), self-perceived health status general (*OR* = 1.29) or bad (*OR* = 1.73), having a history of diabetes (*OR* = 1.45) and CHD (*OR* = 1.89), drinking alcohol (*OR* = 1.51) and exercise (*OR* = 1.10). Protective factors were identified as working in eastern China (*OR* = 0.84), socioeconomic development level medium (*OR* = 0.89) or low (*OR* = 0.83), sleep quality general (*OR* = 0.76) or good (*OR* = 0.65).

In nonphysical violence, influencing factors were consistent with those associated with any type of WPV, and more detailed information has listed in Table 4.

## Discussion

The investigation has shown that the prevalence of any type of WPV, physical violence, and nonphysical violence in a large sample of Chinese ED nurses were 79.39%, 39.65%, and 78.38% respectively. And the reported prevalence was higher than Italian [22], Egypt [23] and Jordan [24], but was lower than Oman [25], Saudi Arabia [26] and Pakistan [27], which indicates a relatively high level of WPV against ED nurses in China. This high level of workplace violence, especially physical violence, may cause tremendous injuries to ED nurses, and indicates a lack of security guards and preventive measures, that need to improve [28].

The majority of perpetrators we discovered was patients’ relatives and gender was male, and the most frequent period was night shift. Patients’ relatives generally had direct contact with ED nurses on behalf of the patients because of patients’ emergency and critical illnesses, which caused frequent frictions [29,30]. The perpetrators were mainly male, since males were more aggressive than females, several studies had shown similar characteristics [31,32]. Violence occurs mainly at night, fewer nurses at night and unable to meet patients’ needs in time can be the reason [33,34].

Apart from male gender, bachelor’s degree, low average monthly salary, shift work and high work stress, had been proved as risk factors in previous studies [17,22,28,33,35]. ED nurses who were older, had higher professional titles and had more work experience, in our research, had a higher risk of WPV, which is contrary to previous studies [28,36,37]. And this might relate to the work characteristics of ED nurses in China, less-experienced ED nurses in large general hospitals took easier scheduling and had fewer workloads, while senior ED nurses tend to perform more complicated work [38,39].

In terms of geographic region, eastern China has the best control of all three types of violence. In China, quality medical resources of provincial and city-scale with high levels are concentrated in the east of Hu Line [40], which means better medical resources were concentrated in eastern China. High-quality medical resources indicates better medical services and treatment effects for patients, better human resource management which could provide more psychological support to ED nurses, and multiple feedback channels for patients, which was of great significance to reduce the risk of WPV [41]. In spite of the factors mentioned above, higher income level, better health insurance reimbursement, higher patients’ medical literacy and education level as the characteristics of well-developed regions, might all associate with lower risk of WPV.

However, in terms of socioeconomic development level in this study, low-developed regions were less likely exposed to all three types of WPV. According to China Statistic Yearbook-2018, high-developed regions had more health care facilities with high-quality medical resources, more patients came here for a better outcome which leads to high tension in patients’ hospitalization [6,31,40]. Crowded environments, staff shortage and long waiting times, common in high-pressure hospitals, will increase the incidence of WPV, as have been verified in previous studies [33,42].

Moreover, ED nurses’ diseases history, especially the history of CHD, had a positive prediction of WPV, while good habits had a negative prediction. As for the history of CHD, it might relate to the high workload. In previous studies, high workload will significantly reduce work efficiency and result in poor outcomes [17,43]. Besides, ED nurses with CHD had more negative attitudes and poorer life quality than healthy ones, which also have been proved as risk factors for WPV [44]. And good habits associated with a more positive attitude toward life, and healthier lifestyle can improve work outcomes which will decrease the risk of WPV [45].

Furthermore, Chinese ED nurses still fail to take actions to protect themselves even after WPV, and the most important one is to submit a violence report. According to previous studies, submitting violence reports is of great importance in improving organizational management and obtaining psychological support, which will benefit all staff in the emergency departments [19,46]. In addition, the top three reasons for WPV were unmet patients’ need, long waiting times, and alcohol/drug abuse. Therefore, violence preventive training for ED nurses, reasonable emergency triage for patients and target hardening of infrastructure including installing security cameras and security guards will have a positive effect on WPV prevention [3,12].

### Strengths and Limitations

This is the first investigation of the prevalence of WPV and the relevant determinants among ED nurses in China at a national level. Secondly, the large sample size significantly increases the statistical power to identify the predictors of WPV against ED nurses. Finally, data collection through universal social networks has greatly improved the response rate of the questionnaire, and reduced the survey bias, which makes the survey results have promotion significance.

Some limitations should be acknowledged in our research. Firstly, this is a cross-sectional survey, and the causal relationship between variables cannot be established; therefore, further longitudinal studies are needed. Secondly, the data was obtained by self-report questionnaire, and the respondents inevitably had recall bias, which may overestimate the outcome. Thirdly, potential factors for WPV against ED nurses are more than listed in the questionnaire, and we cannot identify all of them.

### Implications for Research and Practice

This research is a large-scale cross-sectional study at the national level, revealing the incidence and predictors of WPV against ED nurses in China. However, the following aspects can be improved. First, the problems of horizontal violence among nurses, such as bullying and discrimination, were not given attention in this study. Besides, we observed differences in the risk of WPV against ED nurses among geographic regions and socioeconomic development levels, but these differences deviate from the socioeconomic level across geographic regions in China. Therefore, expanding the factors related to the hospitals’ environments and medical resources are recommended to understand the specific mechanism. Finally, longitudinal studies can be conducted to clarify the causal relationship between variables.

For policymakers, this study found that the popularization of violence preventive training for ED nurses and target hardening of infrastructure in EDs were of great significance to reduce the incidence of WPV. Conducting a rational emergency triage and arranging on-call experienced nurses during night is essential for WPV prevention. Moreover, this result showed that a considerable proportion of ED nurses who experienced WPV took no action; thus, it is important for hospitals to promote violence reporting process and improve follow-up WPV intervention support in China.

### Conclusion

The prevalence of WPV against ED nurses was relatively high than other countries, and the high prevalence of WPV displays the higher workload and bad work environment of ED nurses. Risk factors were identified as: male nurses, bachelor’s degree, average monthly salary between 5,001 and 12,000, working in central China, intermediate and senior professional title, work tenure greater or equal to 10 years, shift work, medium or high work stress, self-perceived health general or bad, history of CHD and alcohol drinking; while protective factors were identified as: working in eastern China, medium or low socioeconomic development level, exercising, and general or good sleep quality. Taking steps to increase the violence reporting rate and improving the work environments will reduce the prevalence of WPV effectively.
